# Association between periodontal diseases and COVID-19 infection: a case–control study with a longitudinal arm

**DOI:** 10.1007/s10266-023-00797-x

**Published:** 2023-03-03

**Authors:** Aysegul Sari, Nursel Kaya Dikmen, Luigi Nibali

**Affiliations:** 1 Faculty of Dentistry, Department of Periodontology, Mustafa Kemal University, Hatay, Turkey; 2 Faculty of Dentistry, Periodontology Unit, Centre for Host-Microbiome Interactions, Oral and Craniofacial Sciences, Kings College London, London, UK; 3 Faculty of Dentistry, Department of Chest Diseases, Mustafa Kemal University, Hatay, Turkey

**Keywords:** Periodontal disease, Periodontitis, COVID-19, Cytokine storm

## Abstract

Some studies have suggested potential relationships between periodontal disease and COVID-19, explained by many possible pathological pathways. The aim of this case–control study with a longitudinal arm was to investigate this association. 80 systemically healthy individuals (apart from COVID-19) were involved in this study, divided into 40 patients who had recently had COVID-19 (test, divided into severe and mild/moderate cases) and 40 who had not had COVID-19 (control). Clinical periodontal parameters and laboratory data were recorded. Mann–Whitney *U* test, Wilcoxon test, and chi-square test were performed to compare variables. Multiple binary logistic regression method was used to estimate adjusted ORs and 95% confidence interval. Hs-CRP-1 and 2, Ferritin-1 and 2, lymphocyte count-1 values, and neutrophil/lymphocyte ratio-1 were higher in patients with severe COVID-19 than patients with mild/moderate COVID-19 (*p* < 0.05). All of these laboratory values significantly decreased after COVID-19 treatment (*p* < 0.05) in the test group. Presence of periodontitis (*p* = 0.015) was higher and periodontal health was lower (*p* = 0.002) in the test group than in the control group. All clinical periodontal parameters were significantly higher in the test group than in the control group (*p* < 0.05), except plaque index. Prevalence of periodontitis was associated with increased odds of having COVID-19 infection (PR = 1.34; 95% CI 0.23–2.45) in the multiple binary logistic regression. COVID-19 is associated with periodontitis prevalence, through a series of possible mechanisms including local and systemic inflammatory responses. Further studies should investigate whether the maintenance of periodontal health may be a factor in the reduction of the severity of COVID-19 infections.

## Introduction

Coronavirus SARS-CoV-2, a member of the Coronaviridae family, caused a COVID-19 disease outbreak in 2019 [1]. While the majority of patients with COVID-19 infections had mild symptoms, around 2% of COVID-19 cases resulted in mortality, intensive care units were necessary for 5%, and hospitalization was observed in 14% of affected individuals [2, 3].

Severe cases of COVID-19 can cause multiple organ failure by complications including septic shock, sepsis, and acute respiratory failure [4]. This is induced by the excessive release of proinflammatory cytokines, termed the cytokine storm, leading to widespread tissue damage characterized by the excessive immune response of the host [4]. COVID-19 severity is associated with systemic diseases, such as cardiovascular diseases, chronic lung and kidney disease, liver disease, asthma, obesity, and diabetes. Also, poor oral health is estimated to have an impact on the risk of developing COVID-19 [5]. COVID-19 infections could be associated with periodontal disease, a chronic inflammatory disease of the supporting apparatus of the teeth. The relationship between COVID-19 and periodontal diseases may be linked with common risk factors, such as obesity, diabetes mellitus, cardiovascular disease, obesity, age, and smoking, which play a role in the severity of both diseases [6, 7]. Manifestations of COVID-19 infection, such as ulcers and erosions [8], white-red plaques [9], post-inflammatory pigmentation [9], petechia [9], erythema multiforme-like lesions [10], and necrotizing periodontal disease [11], have been observed in the mouth. The gingiva is among the most common sites of involvement in the mouth [12].

Angiotensin Converting Enzyme II (ACE2) is shown as a pathway that refers to the relationship between COVID-19 and periodontal disease. Co-expression of ACE2 and transmembrane serine protease 2 is required for SARS-CoV-2 to enter the human body via the oral mucosa [13]. ACE2 is highly secreted from salivary glands and oral epithelial cells [14, 15]. At the same time, this virus uses the spike protein to bind to CD147 to infect human cells, and this protein is highly secreted by epithelial cells in the oral mucosa [16, 17]. In addition, the detection sensitivity of gingival sulcus fluid (GCF) was found to be 63.64% compared to nasopharyngeal swabs [18]. The finding that SARS-CoV-2 is detectable in gingival crevicular fluid (GCF) supports the hypothesis that poor oral hygiene can increase viral load in the oral cavity [19].

The similar overexpression of several cytokines is indicated as one of the pathways for the relationship between both diseases. During the cytokine storm that occurs in COVID-19 infection, serum levels of IL-17 and IL-6 rise [3]. SARS-CoV-2 has elevated Th17 pathway responses. The inflammatory response of the Th17 pathway has been implicated in the pathology of COVID-19 virus-induced lung infections by causing cytokine storm [20]. Interstitial pneumonia seen in severe COVID-19 infections is induced by excessive IL-6 secretion [21]. These cytokines play an important role in the pathogenesis of periodontal disease [22]. In addition, periodontal disease contributes to the increase of systemic inflammation by generating cytokine storm in the presence of other chronic dysmetabolic diseases such as diabetes [23, 24].

Despite some initial studies, the relationship between COVID-19 and periodontal diseases has yet to be confirmed.

Whether periodontal status affects the excessive immune response in individuals exposed to cytokine storms has not been clear. Our null hypothesis is that there is no difference in the prevalence of COVID-19 infection between patients with and without periodontitis. The aim of this study is to evaluate a potential association between periodontal disease and COVID-19.

## Methods

### Study population

The study included two parts as case–control and prospective design. The study was carried out as a joint collaboration between the Department of Chest Diseases in the Faculty of Medicine and the Department of Periodontology in the Faculty of Dentistry at Hatay Mustafa Kemal University, Hatay, Turkey during the period from May 2021 to December 2021. The study protocol was approved by the Ethics Committee for the Use of Human Subjects in Research of Hatay Mustafa Kemal University (Protocol No: 2021/49; 06.05.2021), and the study was carried out in accordance with the tenets of the Declaration of Helsinki. The case–control part of the study conforms to Strengthening the Reporting of Observational Studies in Epidemiology guidelines for observational studies [25].

80 systemically healthy individuals (apart from COVID-19) were involved in this study. The study protocol was explained. Written informed consent was received from each individual prior to enrollment. The study population included 40 patients who had COVID-19 (test) and 40 individuals (controls) who did not have COVID-19. All participants underwent a Rt-PCR test and were recruited from among patients referred to the Department of Chest Diseases, Faculty of Medicine. Patients with previous COVID-19 diagnoses had been followed up at the Department of Chest Diseases, while controls were systemically healthy participants who attended for routine check-ups and had no previous known history of COVID-19 infection. A total of 85 patients were screened to recruit 40 test patients fitting inclusion/exclusion criteria. Following this, a total of 225 individuals were screened to recruit 40 consecutive controls sex-matched to test patients based on the study inclusion/exclusion criteria.

The inclusion criteria for all participants were as follows: a) at least 18 years of age, b) at least 18 teeth present, c) no periodontal treatment in the last 6 months, d) no systemic antibiotic therapy in the last 3 months. Exclusion criteria for all participants were as follows: (a) participants who had self-reported previous systemic disease, such as diabetes or cardiovascular disease, (b) undergone immunosuppressive therapy, (c) were pregnant and/or breastfeeding. For test patients, a further inclusion was previous diagnosis of COVID-19 (within 3–6 months), while exclusions were intubation and systemic steroid therapy. For controls, exclusion was diagnosis of any respiratory disease. After their formal written consent, their medical history, demographics, clinical, laboratory, and radiological data were collected from the medical records, including chest computed tomography scans and previous and current treatments. Also, data on the following characteristics were collected: frequency of dental check-up, tooth brushing frequency, and smoking status. Frequency of dental check-up was defined as ‘regular’ for patients attending the dentist at least every 6 months and ‘irregular’ for those attending less often.

Test patients were subdivided based on their symptoms, as recorded in their previous assessment when first seen in the Department of Chest Diseases: (i) Severe: including patients with symptoms, such as fever, muscle/joint pain, cough, sore throat, tachypnea (30/min), oxygen saturation (SpO2) level < 90% in room air, and bilateral diffuse pneumonia findings on chest radiograph or computed tomography; (ii) Mild–moderate: patients with symptoms, such as fever, muscle/joint pain, cough and sore throat, respiratory rate < 30/minute, SpO2 level in room air > 90%, and no signs of pneumonia in chest X-ray or tomography. These patients had the disease without a history of hospitalization.

### COVID treatment protocol

Treatment for patients diagnosed with COVID-19 followed these protocols:

- Mild–moderate COVID-19 patients received symptomatic treatment for fever and pain, such as antipyretics, adequate nutrition, and appropriate rehydration.
- Severe COVID-19 patients received antiviral therapy (favipiravir). Close saturation monitoring and single-use, disposable oxygen-conducting interfaces (nasal cannula, Venturi mask, and mask with reservoir bag) were provided with oxygen support for these patients. Intravenous fluid (iv liquid) support was applied to patients with impaired oral intake. Pharmacological prophylaxis, such as low molecular weight heparin (such as enoxaparin), according to local and international standards, to prevent venous thromboembolism, when not contraindicated, was also used. These patients were in hospital for an average of 5–7 days.

### Laboratory parameters

The patients' laboratory data included high-sensitivity C-reactive protein levels (hs-CRP) (mg/L), ferritin values (ng/ml), white blood cell count (WBC) (× 10^3^ µL), lymphocyte count (LYM) (× 10^3^ µL), neutrophil count (× 10^3^ µL), and neutrophil/lymphocyte ratio (NLR) (%). Blood samples were collected from the patients into tubes containing EDTA for hemogram. Complete blood count analyses were carried out on a hematological auto analyzer device (Mindray BC 6000, Shenzhen, China) within one hour of the collection of samples. All data presented were obtained from the Department of Chest Diseases in the Faculty of Medicine at Hatay Mustafa Kemal University, Hatay. The patients' laboratory data were obtained at two time points, first on presentation at the hospital before COVID-19 treatment (timepoint 1) (for example, CRP-1). The laboratory data obtained concomitantly to periodontal assessment were defined as timepoint 2 (for example, CRP-2).

### Clinical periodontal parameters

Upon confirmation of eligibility for enrollment in the study, clinical periodontal measurements including probing pocket depth (PPD) (mm), clinical attachment loss (CAL) (mm), plaque index (PI) [26], gingival index (GI) [27], and bleeding on probing (BOP) (presence/absence) (%) [28] were recorded from all participants (test and control) during their visit to the Periodontology Department at timepoint 2. Clinical periodontal measurements were performed at six sites on each tooth (mesio-buccal, mid-buccal, disto-buccal, mesio-lingual, mid-lingual, and disto-lingual locations), except for third molars, using a manual periodontal probe (Williams, Hu-Friedy, Chicago, IL, USA) by a single trained examiner (AS). Intra-examiner agreement was determined for CAL. The intra-examiner reproducibility was determined through repeated examinations of 10 subjects with a one-hour interval (k = 0.95).

Diagnosis of periodontal disease was based on clinical and radiographic criteria proposed by 2017 World Workshop on the Classifications of Periodontal and Peri-implant Disease and Conditions [29]. Individuals with a BOP < 10% without attachment loss and radiographic bone loss were considered to have periodontal health [30]. Individuals presenting with a BOP ≥ 10%, and PPD ≤ 3 mm without attachment loss and radiographic bone loss were considered gingivitis [31]. The criteria for patients with periodontitis were (1) interdental CAL detectable at ≥ 2 non-adjacent teeth or (2) buccal or oral CAL ≥ 3 mm with PPD > 3 mm detectable at ≥ 2 teeth [32]. The periodontal examiner was not blind to the test or control status.

### Statistical analysis

The primary outcome of the study was the difference in the presence and severity of COVID-19 infection. Secondary outcomes were periodontal clinical measurements and laboratory parameters at both timepoints in test sub-groups (patients with mild-moderate vs. severe COVID-19). G-power version 3.1.9 was used for sample size calculation. The sample size calculation was performed based on OR = 6.34 and 43% expected periodontitis prevalence in mild COVID-19 group based on a previously published study [33]. The minimum required sample size was calculated as 36 for each group (α = 0.05, 1-β = 0.95). Considering dropouts, the required number of participants for each group was determined as 40.

The normality of distribution of continuous variables was tested by ShapiroWilk test. Mann–Whitney *U* test was used to compare non-normal numerical data between test and controls. Wilcoxon test was applied for before and after comparison of non-normal data. The relationship between categorical variables was tested using chi-square test, and Bonferroni correction was performed for pairwise comparison. Median and interquartile ranges were given as descriptive statistics. Spearman rank correlation was used to investigate the relationship between non-normal numerical variables.

Multiple binary logistic regression method was used to estimate adjusted ORs and 95% confidence interval. VIFs (variance inflation factor) were evaluated to avoid multicollinearity. The prevalence odds ratio was calculated as the prevalence ratio (PR). Statistical analysis was performed with SPSS for Windows version 24.0, and a p value < 0.05 was accepted as statistically significant.

## Bullet results

### Demographic findings

38 males and 42 females individuals aged 23 to 64 were included in the study.

Table 1 shows characteristics of populations of study. Sex, age, and body mass index (BMI) were similar between the groups (*p* = 1.00, *p* = 0.485, and *p* = 0.791 respectively). Smoking and dental check-up frequency were similar between the groups (*p* = 0.576 and *p* = 0.237 respectively). Tooth brushing frequency was higher in test group than control group (*p* = 0.002). None of the patients had been to the dentist in the last six months.

**Table 1:** Demographic variables of test and control groups

Variables	Test group (*n* = 40)	Control group (*n* = 40)	*P *value
Age median (IQR: 25–75)	47 (31.5–55)	44 (36–52)	0.485
Bmı median (IQR: 25–75)	24.22 (22.94–28.2)	24.4 (23.37–25.83)	0.791
Tooth brushing frequency daily (IQR: 25–75)	2 (1–3)	1 (1–2)	0.002
*Sex* *n* (%)			
Male	19 (47.5)	19 (47.5)	1.000
Female	21 (52.5)	21 (52.5)	
*Frequency of dental check-up* *n* (%)			
Regularly	11 (27.5)	16 (40)	0.237
Unregularly	29 (77.5)	24 (60)	
*Smoking* *n* (%)			
Yes	9 (22.5)	7 (17.30)	0.576
No	31 (77.5)	33 (82.5)	

^*^Statistically significant at p < 0.05

*P* value was obtained using chi-square test for categorical variables and Mann–Whitney *U* test for numerical variables

*BMI* Body mass index

### Periodontal clinical findings

Table 2 reports periodontal disease prevalence and periodontal clinical parameters in the study groups. A higher prevalence of periodontitis compared with gingivitis and periodontal health was detected in the test group (*p* = 0.003). In line with this, all clinical periodontal parameters related to periodontal disease severity were higher in the test group than in the control group. In particular, GI, BOP (%), PD, CAL, and the number of missing teeth were statistically significantly higher in the test group than in the control group (*p* < 0.05), except PI, which did not reach statistical significance (*p* = 0.052).

**Table 2:** Comparison of clinical periodontal parameters between test and control groups

Variable		Test group (*n* = 40)	Control group (*n* = 40)		*P *value
Periodontitis		17 (42.5)	7 (17.5)	RR (95% CI) 2.42 (1.13–5.21)	0.0227*
Non-Periodontitis		23 (57.5)	33 (82.5)	1 (reference)	
Prevalence of periodontal	PH	4 (10)^†^	16 (40)		
Status	G	19 (47.5)	17 (42.5)		0.003*
*n* (%)	P	17 (42.5)^†^	7 (17.5)		
PI		1.33 (0.98–1.99)	1 (0.21–1.68)		0.052
GI		1.36 (0.94–1.68)	1.17 (0.18–1.57)		0.038*
BOP (%)		42.18 (23.44–69.41)	21.51 (2.68–56.94)		0.027*
PD (mm)		2.52 (1.95–3.01)	1.75 (1.52–2.82)		0.008*
CAL (mm)		2.54 (2.04–3.01)	1.75 (1.57–2.82)		0.006*
Number of missing teeth		2 (0–5)	0.5 (0–2.5)		0.031*

^*^Statistically significant at P < 0.05

*P* value was obtained using chi-square test for categorical variables and Mann–Whitney *U* test for numerical variables

Bonferroni correction was performed for pairwise comparison of categorical variables

Data are expressed as median and 25% to 75%

^†^*P* < 0.05 versus Control Group *PH* periodontally Health, *G* Gingivitis *P* Periodontitis

*PI* plaque index, *GI* gingival index, *BOP* percentage bleeding on probing, *PD* probing pocket depth, *CAL* clinical attachment level

### Laboratory findings

Table 3 shows the laboratory parameters of COVID-19 sub-groups. Fifteen patients attended the study appointment 3 months post-COVID hospital admission, while 25 patients attended between 4 and 6 months post-COVID hospital admission. The average period was 4.56 ± 2.12 months between COVID-19 infection and the study visit. Laboratory parameters that increase with the presence of COVID-19 infection were higher in patients with severe COVID-19 than patients with mild/moderate COVID-19. Also, they decreased after COVID-19 treatment. Hs-CRP-1 and 2, Ferritin-1 and 2, LYM-1 values, and NLR-1 were higher in patients with severe COVID-19 than patients with mild/moderate COVID-19 (*p* = 0.001, *p* = 0.028, *p* = 0.002, *p* = 0.010, *p* = 0.001, *p* = 0.006). Hs-CRP, Ferritin, LYM values, and NLR decreased after treatment in patients with COVID-19 (*p* = 0.001, *p* = 0.001, *p* = 0.001, *p* = 0.012, respectively).

**Table 3:** Comparison of clinical laboratory parameters between and within COVID-19 sub-groups

	Severe (*n* = 15)	Mild/moderate (*n* = 25)	*P*
CRP-1 (mg/L)	73.2 (29–172)	27.2 (14.2–36)	0.001*
CRP-2 (mg/L)	4.52 (3.64–9.2)	3.13 (3.13–3.3)	0.028*
	*P* = 0.001	*P* = 0.001	
Ferritin-1 (ng/ml)	215 (180.9–858)	159.6 (56.4 -203.6)	0.002*
Ferritin-2 (ng/ml)	58.2 (37.4–201)	30.5 (16.4–60)	0.010*
	*P* = 0.001	*P* = 0.001	
WBC-1 (× 10^3^ µL)	6.7 (5.8 -10.98)	7.2 (6.2–8.57)	0.783
WBC-2 (× 10^3^ µL)	7.58 (6.74 -12.01)	7.38 (6.45–8.17)	0.267
	*P* = 0,078	*P* = 0.861	
LYM-1 (× 10^3^ µL)	1.17 (0.91–1.4)	1.96 (1.24–2.46)	0.001*
LYM-2 (× 10^3^ µL)	2.71 (1.94 -2.82)	2.39 (2.13–2.64)	0.05
	*P* = 0.001	*P* = 0.05	
Neutrophil-1 (× 10^3^ µL)	4.86 (4.39–9.21)	5,12 (3.78 to – 6.11)	0.472
Neutrophil-2 (× 10^3^ µL)	5.19 (3.94 -9.51)	4.55 (3.65 to – 5.15)	0.119
	*P* = 0.909	*P* = 0.032	
NLR-1 (%)	5.17 (3.75–6.58)	2.79 (1.69–4.42)	0.006*
NRL-2 (%)	1.7 (1.43 -3.51)	1.9 (1.52–2.25)	0.890
	*P* = 0.012	*P* = 0.001	

^*^Statistically significant at *P* < 0.05

*P* value was obtained using Mann–Whitney *U* test for between groups and Wilcoxon test for within groups

Data are expressed as median and 25% to 75%

*CRP-1* C-reactive protein during COVID-19, *CRP-2* C-reactive protein post-COVID-19, *WBC-1* white blood cell during COVID-19, *WBC-2* white blood cell post-COVID-19, *LYM-1* lymphocyte during COVID-19, *LYM-2* lymphocyte during COVID-19

*NLR-1* neutrophil/lymphocyte ratio during COVID-19, *NRL-2* neutrophil/lymphocyte ratio post-COVID-19

Table 4 shows correlations between periodontal and laboratory parameters in test patients. Positive correlations were detected between number of missing teeth and hs-CRP-1, WBC-2, neutrophil-2 values, and NLR-2 (*p* ≥ 0.05). There was a positive correlation between PI and hs-CRP-1 values, and GI and hs-CRP-2 values, while PPD and CAL did not show correlations with laboratory parameters.

**Table 4:** Correlations between periodontal and laboratory parameters in test group

Veriables*		PI	GI	BOP	PPD	CAL	Number of missing teeth
hs-CRP-1	*r*	0.332^*^	0.284	0.250	0.043	0.103	0.423^**^
	*P*	0.036	0.076	0.119	0.792	0.527	0.006
hs-CRP-2	*r*	0.282	0.329^*^	0.306	0.005	0.038	0.269
	*P*	0.078	0.038	0.055	0.976	0.815	0.093
WBC-2	*r*	0.083	0.179	0.252	0.016	– 0.031	0.482^**^
	*P*	0.609	0.270	0.116	0.920	0.849	0.002
Neutrophil-2	*r*	0.150	0.244	0.262	– 0.028	0.024	0.515^**^
	*P*	0.355	0.129	0.102	0.863	0.884	0.001
NLR-2	*r*	-0.107	0.044	0.030	– 0.095	– 0.062	0.332^*^
	*P*	0.510	0.787	0.853	0.562	0.706	0.036

^*^Statistically significant at *P* < 0.05 **Statistically significant at *P* < 0.01

*r* Spearman correlation coefficient; *P* value was obtained usingvid Spearman correlation test

*CRP-1* C-reactive protein during COVID-19, *CRP-2* C-reactive protein post-COVID-19, *WBC-2* white blood cell post-COVID-19

*NRL-2* neutrophil/lymphocyte ratio post-COVID-19

### Multiple binary logistic regression model

Table 5 reports the multiple binary logistic regression models. It was performed to ascertain the effects of presence of periodontitis, sex, smoking status, age, and BMI on the likelihood that participants had COVID-19 infection. Prevalence of periodontitis was associated with increased odds of having COVID-19 infection as independent confounding factor (PR = 1.34; 95% CI 0.23–2.45) in the multiple binary logistic regression.

**Table 5:** Multiple binary logistic regression for affecting factor of COVID-19 infection

Variable	Test group (*n* = 40)	Control group (*n* = 40)		
Group	*N* (%)	*N* (%)	PR (95% CI)	*P*
Periodontitis	17 (42.5)	7 (17.5)	1.34 (0.23–2.45)	0.018*
Non-periodontitis	23 (57.5)	33 (82.5)	1 (reference)	N/A
*Sex*				
Male	19 (47.5)	19 (47.5)	0.18 ( – 0.75 to – 1.13)	0.697
Female	27 (52.5)	27 (52.5)	1 (reference)	N/A
*Smoking*				
Yes	9 (22.5)	7 (17.5)	0.01 ( – 1.21 – 1.2)	0.991
No	31(77.5)	33 (82.5)	1 (reference)	N/A
	Median (%25–%75)	Median (%25-%75)	PR (95% CI)	P
Age	47 (31.8–55)	44 (36–52)	0.02 ( – 0.06 to – 0.03)	0.516
BMI	24.22 (22.96–28.41)	24.4 (23.38–25.68)	0.08 ( – 0.06 to – 0.21)	0.277

^*^Statistically significant at *p* < 0.05, *CI* confidence interval, *N/A* not applicable

*PR* Prevalence ratio, *BMI* Body mass index

## Discussion

This is one of the first clinical studies to evaluate the association between COVID-19 and periodontal disease. The findings of the present study show an association between periodontal clinical parameters and COVID-19 infection. Periodontitis prevalence and all clinical periodontal parameters were increased in patients post-COVID-19 compared with healthy controls, independent of sex, age, BMI, smoking, and dental check-up frequencies [34–38]. The adjustment for these potential confounders is important, since it has been reported in a previous study that the possible relationship between COVID-19 and periodontal diseases may be related to the risk factors shared by both of these diseases [6]. For this reason, care was taken to ensure that these possible confounding factors were similar between the groups and not affecting the associations. Similarly, the frequency of dental check-up, which could affect periodontal parameters, was not different between groups [39].

Many inflammatory markers are elevated during COVID-19 infection. Inflammatory markers, such Hs-CRP, Ferritin, LYM, and NLR, are important indicators used both to show the severity of COVID-19 and to evaluate the prognosis of the disease [40–42]. A study reported that 2601 (93.5%) of 2782 COVID-19 patients over the age of 18 years had high Hs-CRP rates and in the presence of CRP found above the median value, more severe infections, such as venous thromboembolism and pneumonia, were found in the COVID-19 patients [40]. In another study, the authors found leukopenia in 33.7%, thrombocytopenia in 36.2%, and lymphocytopenia in 83.2% of patients at the time of admission in 1099 COVID-19 patients. Additionally, they found more pronounced lymphocytopenia and laboratory abnormalities, such as leukopenia, in patients with severe disease compared with those with non-severe COVID disease [41]. NLR calculated simply by the neutrophil count/lymphocyte count ratio elevation is thought to be due to upregulation of genes involved in the lymphocyte cell death pathway, abnormal increase in pathological low-density neutrophils, and dysregulated expression of inflammatory cytokines caused by the SARS-CoV2 infection mechanism [42]. NLR has been determined to be an independent risk factor for COVID-19 disease severity [43]. Cytokine storm occurred when an immune system infection is over-activated by infection, drugs, and/or some other stimuli and is characterized by the release of cytokines into the circulation at high levels [4, 20]. As the cytokine storm is in progress, the amount of lymphocytes decreases dramatically, while acute-phase reactants such as ferritin increase spontaneously [44]. In addition, it has been reported that an excessive increase in serum ferritin levels is associated with hyperferritinemia, high mortality, and admission to the intensive care unit [45]. In accordance with the previous studies, Hs-CRP, Ferritin, LYM values, and NLR were higher in patients with severe COVID-19 than patients with mild/moderate COVID-19 before COVID treatment. Hs-CRP and Ferritin values were higher in patients with severe COVID-19 than patients with mild/moderate COVID-19 after COVID treatment. Hs-CRP, Ferritin, LYM values, and NLR decreased after treatment in patients with COVID-19 in the present study.

The relationship between periodontal disease and COVID-19 has been explained by many possible pathological pathways such as having common risk factors, having similar cytokine pathways in the chronic inflammatory response, an increase in blood biomarkers, such as WBC and hs-CRP in both diseases, and ACE-2 secretion which is required for virus load in oral epithelial cells [3, 6, 14]. A previous study noticed that the COVID-19 pandemic negatively affected oral health and was associated with increased periodontal disease complaints, such as bleeding and pain in the gingival tissue [46]. In the present study, the presence of periodontitis was higher in the post-COVID-19 group than in the control group. Clinical periodontal parameters, such as GI, BOP, PD, CAL, and the number of missing teeth, were higher in the post-COVID-19 group than in the control group. Overall, prevalence of periodontitis was associated with an increased COVID-19 infection (PR = 1.34; 95% CI 0.23–2.45) following adjustment for sex, smoking status, age, and BMI. These findings are in agreement with a few other recent studies [33, 46–48]. Marouf et al. showed that the risk of having COVID-19 complications in patients with periodontitis was OR 4.57 (95% CI 1.19–17.4) for the need of assisted ventilation, 8.81 (95% CI 1.00–77.7) for death, OR of 3.67 (95% CI 1.46–9.27) for all COVID-19 complications after adjusted possible confounding factors in the study with a cross-sectional design evaluating the association between periodontitis and COVID-19 complications [33]. Another study reported that COVID-19 patients with bleeding or painful gums had a higher risk of death during COVID-19 infection (OR = 1.71, 95% CI 1.05–2.72) [47]. Anand et al. reported that the mean CAL and PI score and severe prevalence of periodontitis and gingivitis were higher in post-COVID patients than in controls. In a case–control study, they observed associations between COVID-19 and CAL (OR = 8.46; 95%CI 3.47–20.63), plaque scores (OR = 7.01; 95% CI 1.83–26.94), severe periodontitis (OR = 11.75; 95% CI 3.89–35.49), and gingivitis (OR = 17.65; 95% CI 5.95–52.37) [48]. These findings support a possible relationship between COVID-19 infection and the presence of periodontitis and severity of periodontal disease. As for the direction of association, these case–control studies, including the present study, cannot make any conclusions as to whether the periodontal conditions exacerbate COVID-19 infection or vice versa, or whether other residual confounders contribute to it.

In this study, we also assessed the correlations between laboratory and periodontal clinical parameters in COVID-19 patients. Consistent with the findings of previous studies, we found positive correlations between the number of missing teeth and hs-CRP-1, WBC-2, neutrophil-2 values, and NLR-2 [40–42]. Also, positive correlations were detected between PI and hs-CRP-1 values; and GI and hs-CRP-2 values. During COVID-19 infection, inflammatory markers of blood, such as hs-CRP-1, WBC-2, neutrophil-2 values, and NLR-2 rise in proportion to the severity of COVID-19 infection. These correlations give further support to associations between oral disease and COVID-19, although we are not sure about the reasons for extraction. Similarly, the correlation between GI, PI, and Hs-CRP, which is one of the important inflammatory markers raised in COVID-19, suggests that the severity of periodontal disease may affect COVID-19 infection. On the other hand, it can suggest that Hs-CRP elevation in COVID-19 infection may have a long-term impact on the severity of periodontal diseases through systemic inflammation. In this context, it is important to stress that the clinical assessment of COVID patients was carried out 3 to 6 months post-hospitalization, so it is hard to know whether periodontal conditions deteriorated during or post-COVID-19 infection. However, none of the patients had been to the dentist in the previous 6 months. We need long-term longitudinal evaluations to test this aspect of the relationship.

The major limitations of the present study were the case–control design and relatively small sample size. Other limitations of the study are that data from patients who had mild–moderate and severe COVID-19 with more invasive treatment, which did not include intubation, antiviral and steroid therapy, were combined for the primary outcome and the periodontal examiner was not blind to the test or control status of the participant. In addition, not having the periodontal records and COVID vaccination status of the patients before COVID-19 infection, the evaluation of periodontal status only 3 to 6 months after the COVID-19 infection, and the relatively short follow-up are other limitations of the study. Further studies with larger study populations and longitudinal cohorts are needed to explain the association between periodontal disease and COVID-19 infection.

The present study findings suggest that periodontitis is associated with COVID-19 through a series of possible mechanisms including local and systemic inflammatory responses.

## Data Availability

The datasets analyzed during the current study are available from the corresponding author upon reasonable request.
